# Individual education, area income, and mortality and recurrence of myocardial infarction in a Medicare cohort: the National Longitudinal Mortality Study

**DOI:** 10.1186/1471-2458-14-705

**Published:** 2014-07-09

**Authors:** Sean A Coady, Norman J Johnson, Jahn K Hakes, Paul D Sorlie

**Affiliations:** 1 Division of Cardiovascular Sciences, National Heart, Lung, and Blood Institute, 2 Rockledge Ctr, 6701 Rockledge Dr., Rm10200 MSC 7936, Bethesda 20817 MD, USA; 2 Center for Administrative Records and Research Applications, United States Bureau of the Census, 4600 Silver Hill Road, Suitland 20233 MD, USA

**Keywords:** Myocardial infarction, Epidemiology, Mortality, Socio-economic, Elderly

## Abstract

**Background:**

The Medicare program provides universal access to hospital care for the elderly; however, mortality disparities may still persist in this population. The association of individual education and area income with survival and recurrence post Myocardial Infarction (MI) was assessed in a national sample.

**Methods:**

Individual level education from the National Longitudinal Mortality Study was linked to Medicare and National Death Index records over the period of 1991-2001 to test the association of individual education and zip code tabulation area median income with survival and recurrence post-MI. Survival was partitioned into 3 periods: in-hospital, discharge to 1 year, and 1 year to 5 years and recurrence was partitioned into two periods: 28 day to 1 year, and 1 year to 5 years.

**Results:**

First MIs were found in 8,043 women and 7,929 men. In women and men 66-79 years of age, less than a high school education compared with a college degree or more was associated with 1-5 year mortality in both women (HRR 1.61, 95% confidence interval 1.03-2.50) and men (HRR 1.37, 1.06-1.76). Education was also associated with 1-5 year recurrence in men (HRR 1.68, 1.18-2.41, < High School compared with college degree or more), but not women. Across the spectrum of survival and recurrence periods median zip code level income was inconsistently associated with outcomes. Associations were limited to discharge-1 year survival (RR lowest versus highest quintile 1.31, 95% confidence interval 1.03-1.67) and 28 day-1 year recurrence (RR lowest versus highest quintile 1.72, 95% confidence interval 1.14-2.57) in older men.

**Conclusions:**

Despite the Medicare entitlement program, disparities related to individual socioeconomic status remain. Additional research is needed to elucidate the barriers and mechanisms to eliminating health disparities among the elderly.

## Background

Socioeconomic status (SES) has been associated with mortality and incident coronary heart disease [1-3], and the Medicare entitlement program seeks to mitigate some of these SES differentials by providing universal access to healthcare to US citizens aged 65 years or more. However, residing in areas with fewer economic resources may delay care for acute illness or impact the ability to receive routine or follow-up care. Further, despite Medicare’s aspect of universal care, individual level SES may still impact health outcomes among the elderly through a variety of potential social-behavioral pathways such as chronic stress, a lack of social networks, and access to health information. Although socio-economic status has been shown to be related to all-cause mortality regardless of underlying disease [4], a disproportionate share of the disparities in mortality are due to chronic diseases [5]. An estimated 515,000 incident myocardial infarction (MI) cases occur in the US each year and with an average age >65 years, most are among the Medicare eligible population [6]. Approximately 22% of those surviving the initial MI will have a recurrent event and mortality within the first year after an MI is as high as 30% [6]. Thus, furthering our understanding of the role of area and individual SES on survival and recurrence post MI among Medicare beneficiaries may have significant public health impacts.

Several international studies in locales with universal health care have found significant associations between area and/or individual socioeconomic traits with survival post MI [7-12]. Studies in North America have been less conclusive. In a large Medicare population, significant but modest associations were found for 30 day and 1 year mortality for beneficiaries residing in zip codes with the lowest median income [13], and in a Chicago based Medicare population, Medicaid recipients (defined as higher poverty status) in higher income zip codes did not have a survival benefit, while non-Medicaid recipients in zip codes with higher income had a lower mortality risk [14].

The National Longitudinal Mortality (NLMS) study was linked to Medicare records from 1991-2001 to assess associations between area income and individual education with mortality and recurrence in men and women 66 years of age or more after a first apparent MI. Mortality risk after an MI is initially high, but gradually improves over time. In the NLMS-Medicare sample cumulative mortality was 18% in the first 28 days, 32% at 1 year, and 56% at five years post MI. We hypothesized that differential associations may exist with respect to time since the index MI and that these associations may be further differentiated by sex or age. Factors associated with the area may exert a stronger influence on survival in the short term while individual factors may have a more pronounced association on longer term survival. In order to examine associations with both short and long term survival and recurrence, survival time was partitioned into 3 periods: in-hospital, discharge to 1 year, and 1 year to 5 year follow-up periods, and follow-up for recurrence was partitioned into 2 periods: 28 day to 1 year, and 1 to 5 years. Area SES was defined using the median income in the beneficiary’s home zip code and individual SES was based on attained education.

## Methods

### Data sources

The NLMS is a prospective study of mortality occurring in combined samples of the non-institutionalized United States population drawn from the Census Bureau Annual Social and Economic Supplement (ASEC) ascertained in 1973 and annually from 1978-1998 [15]. Each survey sample is a complex, national probability sample of households surveyed to obtain demographic, economic, and social information about the U.S. population. During the household interview, information was collected on each occupant regarding age, sex, race, Hispanic status, educational attainment, and other socio-demographic characteristics.

Mortality follow-up information was collected through a computer match to the National Death Index (NDI) through 2002. The NDI, a national file containing information collected from death certificates, is maintained by the National Center for Health Statistics. The matching of records to the NDI has been shown to be an effective and accurate means of ascertaining mortality information using personal identifiers including: social security number, name, date of birth, sex, race, marital status, state of birth, and state of residence [15,16]. Each record match to the NDI is classified as either a true match, false match, or questionable match. All questionable matches are adjudicated by hand by comparing information on the death certificate to the matched ASEC record.

Morbidity and health care encounter data was obtained through record-linkage to the Centers for Medicare & Medicaid Services (CMS) Medicare database. All NLMS persons with suitable identifiers were linked to Medicare data from 1991-2001. Of 458,772 NLMS persons that were age and survival eligible for Medicare and for which ASEC sample design information was available, 391,594 (85%) were successfully linked. Those that were not linked to Medicare were similar to those linked to Medicare with respect to sex (56.0% female vs 56.7%), but were slightly younger (61.6 years of age on 1/1/1991 compared with 62.9 years of age), more likely to have an education level less than high school (41.2% vs 36.4%), more likely to have a family income of less $10 K in 1990 dollars (26.6% vs 16.6%), less likely to be married at time of survey (65.6% vs 71.1%), more likely to be black (12.4% vs 8.2%) and more likely to be Hispanic (6.1% vs 3.9%). Since linkage to Medicare was related to SES, a sensitivity analysis was conducted in which the Medicare population was re-weighted adjusting for the probability of Medicare linkage. Medicare linkage probabilities were adjusted by sex, race, ethnicity, family income and marital status. Since the re-weighting was dependent on factors associated with Individual education, original weights will be the focus of the analysis. Enrollment information, hospital admissions, outpatient and physicians visits were extracted for each linked individual. Eligibility for the current study consisted of first hospitalizations with a primary or secondary discharge diagnosis code of 410 according to the ninth revision of the International Classification of Diseases Clinical Modification (ICD-CM 9), and continuous Medicare type A and B coverage for one year prior to the index MI. A primary or secondary discharge diagnosis code of 410 has been shown to have 94% positive predictive value (PPV) for identifying MIs in a Medicare population [17] and a PPV of approximately 75% in the Atherosclerosis Risk in Communities study [18]. Since the year prior to the index MI was utilized for health related covariates, the minimum age for an MI was 66 years and the earliest date for an index MI was January 1, 1992. Exclusions included admissions from skilled nursing facilities, transfers from other health care facilities, and hospitalizations lasting more than 30 days. To isolate incident cases, ICD-CM9 discharge codes 410.*x*2 were excluded as an indication of a repeat encounter, and persons with any mention of ICD9 codes 410 or 412 (old myocardial infarction) among the physician encounters, outpatient and hospital records for one year prior to the index MI were excluded.

### Covariates

Procedure codes at the index hospitalization were searched for mention of coronary revascularization (Percutaneous Coronary intervention (PCI) 36.00-36.07, Coronary Artery Bypass Grafting (CABG) 36.10 – 3.6.19). Hospitalization, outpatient, and physician visit records for the year proceeding the MI were searched for any mention of a history of CHD (411, 413-414, 429.2), hypertension (401-404), hyperlipidemia (272.0 – 272.4), diabetes (250), or stroke (430 – 438). Sex and race were taken from the ASEC survey as these are self-reported, age in years was calculated from the date of MI and date of birth, and education was categorized into three groups based on the number of years of attained education: less than high school education, high school diploma but no college degree, college degree or more. Area SES was based on the home zip code at time of the index MI as extracted from the hospitalization record. Median income for all US zip codes was extracted from Census Bureau Zip Code Tabulation Areas Summary Tape Files for 1990 and 2000 and divided into quintiles. Zip Code Tabulation Areas are a geographic representation of postal service ZIP code service areas. Infarctions occurring in 1992-1995 were linked to median income quintiles in 1990 and all other MIs were linked to median income quintiles in 2000.

Since the ASEC survey may have occurred 20 years or more prior to enrollment in Medicare, individual measures of SES were limited to education. Due to low numbers, only those self-identifying as non-Hispanic white or black were included in the analysis.

### Outcome definitions

Mortality follow-up was based on the NDI match and recurrence was defined as a subsequent hospitalization with a primary or secondary ICD9 discharge code of 410 or a CHD death defined as an ICD9 underlying cause of death code of 410-414 or ICD10 code of I20-I22, or I24-I25. Any recorded MI within 28 days of the first MI was considered an extension of the first MI and not a recurrent event. Survival follow-up extended through December 31, 2002 while follow-up for recurrence was censored on December 31, 2001. Median follow-up time for survival was 6 years and median follow-up time for recurrence was 5 years.

### Statistical analysis

All analyses were sex specific. To examine potential differential impacts according to follow-up time, survival analyses were conducted in 3 discrete time periods: in-hospital, discharge-1 year, and 1-5 years. Similarly, follow-up for recurrence was separated into 28 day-1 year and 1-5 years. Since all persons had at least one year of follow-up for mortality, log-linear models were used to estimate relative risk associations with in-hospital and discharge-1 year mortality, and proportional hazards regression was used for long term survival and both 28 day-1 year and 1-5 years recurrence. Earlier reports suggested that there may be effect modification by age with respect to SES associations [13]; therefore, analyses were further stratified into age specific groups: 66-79 years and 80+ years. SUDAAN release 10.0.1 was used for all analyses to account for the complex sample design of the ASEC survey.

### Ethics

The ASEC surveys are undertaken to characterize the US civilian, non-institutionalized population in terms of demographics and employment and therefore a traditional informed consent is not utilized. Participation is voluntary and subjects are informed that their data may be linked with other Census data or external data and are provided with the option to opt-out of any data linkage. The NLMS-Medicare study is a data linkage study without any direct interaction with survey respondents, thus the principles of the Helsinki Declaration are not applicable. The Medicare data linkage protocol was reviewed and approved by the Census Data Stewardship Executive Policy group and linkage to NDI further reviewed and approved by the National Association for Public Health Statistics and Information System (NAPHSIS) committee. As part of their review, these committees specifically evaluate the data security practices and participant privacy protections of studies conducting record linkage.

## Results

The final sample included incident MIs from 8,043 women and 7,929 men. As shown in Table 1, the average age at time of MI was 79 years in women and 77 years in men. In both women and men, when compared to the older age group (80+ years), coronary revascularizations at time of MI and a history of hyperlipidemia and diabetes were more likely to be noted among those in the younger age group. Those in the older age group were more likely to have a history of stroke or CHD. While the proportion with a college degree or more were similar by age group, those in the older age group were more likely to have less than a high school education. The MIs in the sample tended to come from higher income zip codes with approximately half of the sample from the top 2 quintiles of median income and approximately one third in the lower 2 quintiles.

**Table 1:** Basic characteristics of apparent first myocardial infarctions (MI) in NLMS persons linked to Medicare by sex and age group

	**Women**						**Men**					
	**All**		**66-79**		**80+**		**All**		**66-79**		**80+**	
	**% or**	**Std**	**% or**	**Std**	**% or**	**Std**	**% or**	**Std**	**% or**	**Std**	**% or**	**Std**
	**Mn**	**Dev**	**Mn**	**Dev**	**Mn+**	**Dev**	**Mn**	**Dev**	**Mn**	**Dev**	**Mn+**	**Dev**
N (Unweighted)	8,043		4,223		3,820		7,929		5,314		2,615	
Age (Years)	79.3	7.7	73.2	3.9	85.9	4.6	76.7	7.0	72.7	3.9	84.9	4.2
Year of Index MI	1996.5	2.9	1996.3	2.9	1996.8	2.9	1996.3	2.8	1996.1	2.8	1996.8	2.8
Followup Time (Years)	6.0	2.9	6.2	2.9	5.8	2.9	6.2	2.8	6.4	2.8	5.7	2.8
Black race	8.1%		9.6%		6.5%		5.4%		5.5%		5.2%	
Death within 1 year	20.8%		13.0%		30.1%		20.2%		15.5%		31.2%	
Recurrent MI (%) (1)	14.0%		10.6%		18.4%		13.5%		11.3%		19.1%	
CABG at MI	4.2%		6.6%		1.5%		7.5%		9.6%		3.1%	
PCI at MI	11.0%		15.8%		5.8%		13.6%		16.6%		7.4%	
Hx of Hypertension	61.6%		60.1%		63.2%		48.2%		47.3%		50.3%	
Hx of Hyperlipidemia	25.3%		30.5%		19.7%		22.1%		24.9%		16.4%	
Hx of Diabetes	30.4%		36.0%		24.4%		26.2%		27.7%		22.9%	
Hx of Stroke	17.2%		15.4%		19.2%		16.1%		13.4%		21.4%	
Hx of CHD	37.4%		34.2%		40.8%		39.3%		36.2%		45.8%	
Education												
< High School	46.2%		42.5%		50.2%		43.3%		40.0%		50.2%	
High School+, but no College Degree	47.0%		51.4%		42.2%		43.4%		46.2%		37.7%	
College degree+	6.8%		6.1%		7.6%		13.3%		13.9%		12.1%	
Zip Code Median Income Quintile												
Q1 (lowest)	13.8%		14.4%		13.1%		11.5%		11.6%		11.1%	
Q2	18.5%		18.0%		19.1%		18.0%		17.6%		18.9%	
Q3	21.1%		21.5%		20.6%		21.2%		21.2%		21.3%	
Q4	22.7%		23.8%		21.6%		23.5%		23.6%		23.4%	
Q5 (highest)	23.9%		22.3%		25.6%		25.8%		26.0%		25.4%	

*Abbreviations: CABG* Coronary Artery Bypass Grafting, *PCI* Percutaneous coronary intervention, *CHD* coronary heart disease.

(1) Recurrent MI or fatal CHD event within 1 year among those surviving at least 28 days.

### Survival

All models were estimated with adjustment for age, race, history of hypertension, hyperlipidemia, diabetes, CHD, Stroke, PCI-CABG at hospitalization, and year of MI. Models were first run including only individual level education, a second run included only zip code level median income quintiles, and finally models were run including both individual level education and zip code median income quintiles. Adjustment for both individual level education and zip code median income quintile only marginally attenuated the risk ratios and hazard rates; therefore, only the fully adjusted results are presented.

As can be seen in Table 2 zip code level median income was not associated with survival in women. The exception, in-hospital mortality in women 66-79 years of age (RR Q1 v Q5 = 1.40; P = 0.037), was not significant in the sensitivity analysis (Additional file 1: Table S1). Individual level education was associated with 1-5 years mortality (HRR: < HS v College Degree + =1.61, P = .035), but not with in-hospital or discharge-1 year mortality. Education and area SES were not associated with survival in women 80+ years.

**Table 2:** Association of education, and zip code median income quintile with in-hospital, discharge to 1 year, and 1-5 year mortality after a first apparent myocardial infarction in women

	**All women**			**Women 66-79 years**			**Women 80+ years**		
	**RR or HRR**	**95% Conf int**	**P value**	**RR or HRR**	**95% Conf int**	**P value**	**RR or HRR**	**95% Conf int**	**value**
In Hospital Mortality									
N (Events)	8,043	(1153)		4,223	(454)		3,820	(699)	
Ed < HS	1.07	(0.84, 1.37)	0.578	0.87	(0.58, 1.31)	0.518	1.18	(0.86, 1.62)	0.307
Ed HS < BS	1.06	(0.83, 1.35)	0.644	0.78	(0.52, 1.18)	0.240	1.25	(0.92, 1.71)	0.156
Zip Inc q1	1.05	(0.86, 1.28)	0.640	*1.40*	*(1.02, 1.92)*	*0.037*	0.85	(0.65, 1.11)	0.245
Zip Inc q2	1.01	(0.84, 1.20)	0.931	1.18	(0.88, 1.60)	0.272	0.93	(0.74, 1.16)	0.511
Zip Inc q3	0.91	(0.76, 1.09)	0.332	0.99	(0.72, 1.36)	0.939	0.88	(0.71, 1.10)	0.268
Zip Inc q4	0.91	(0.76, 1.08)	0.261	0.89	(0.66, 1.20)	0.434	0.94	(0.76, 1.17)	0.596
Discharge - 365 day Mortality									
N (Events)	6,852	(1392)		3,760	(484)		3,092	(908)	
Ed < HS	1.07	(0.87, 1.32)	0.516	1.23	(0.80, 1.89)	0.347	1.03	(0.82, 1.31)	0.779
Ed HS < BS	0.95	(0.77, 1.17)	0.620	0.95	(0.62, 1.47)	0.834	0.97	(0.76, 1.24)	0.824
Zip Inc q1	1.02	(0.86, 1.22)	0.785	0.86	(0.62, 1.20)	0.377	1.10	(0.90, 1.35)	0.344
Zip Inc q2	1.05	(0.90, 1.23)	0.505	0.93	(0.70, 1.24)	0.626	1.10	(0.92, 1.31)	0.291
Zip Inc q3	0.98	(0.84, 1.15)	0.831	0.92	(0.69, 1.22)	0.546	1.01	(0.84, 1.21)	0.898
Zip Inc q4	0.98	(0.84, 1.14)	0.771	0.80	(0.59, 1.09)	0.158	1.07	(0.90, 1.28)	0.444
366 days - 5 year Mortality									
N (Events)	5,460	(729)		3,276	(900)		2,184	(1518)	
Ed < HS	1.19	(0.95, 1.50)	0.138	*1.61*	*(1.03, 2.50)*	*0.035*	0.99	(0.76, 1.30)	0.962
Ed HS < BS	1.08	(0.86, 1.35)	0.531	1.41	(0.92, 2.18)	0.117	0.91	(0.69, 1.20)	0.501
Zip Inc q1	1.00	(0.82, 1.21)	0.986	1.11	(0.83, 1.47)	0.486	0.93	(0.72, 1.21)	0.605
Zip Inc q2	0.95	(0.80, 1.13)	0.576	0.95	(0.73, 1.24)	0.721	0.96	(0.77, 1.21)	0.754
Zip Inc q3	0.94	(0.80, 1.11)	0.474	1.06	(0.83, 1.35)	0.656	0.86	(0.69, 1.08)	0.203
Zip Inc q4	1.01	(0.86, 1.19)	0.919	1.04	(0.82, 1.32)	0.756	1.00	(0.81, 1.25)	0.971

*Abbreviations: RR* Relative Risk (In-hospital and Discharge - 365 day mortality), *HRR* Hazard Rate Ratio (1-5 year mortality), *Ed *< HS Education less than a high school diploma, *Ed* HS < BS At least a high school diploma, but no college degree.

Adjusted for Age, race, hx of hypertension, hx of diabetes, hx of lipidemia, hx of CHD, hx of stroke, and coronary revascularization (PCI or CABG) during hospitalization, year of MI.

Education referent group is college degree or more. Zip code median income referent group is quintile 5 (highest median income).

Italicized associations, confidence intervals, and p-values denote those significant at alpha<0.05.

Similar to women, individual level education was associated with long term survival in men 66-79 years of age (Table 3, HRR: < HS v College Degree + =1.37, P = .017). The lowest quintile of zip code level median income was marginally associated with long term mortality in younger men (HRR Q1 v Q5 = 1.30; P = 0.052) in both the primary analysis and in the sensitivity analysis (Additional file 1: Table S2). Although zip code level median income was associated with discharge-1 year mortality among men 80+ years of age, these associations were not significant in the sensitivity analysis. No association was found between individual level education and short or long-term mortality in men 80+ years of age.

**Table 3:** Association of education, and zip code median income quintile with in-hospital, discharge to 1 year, and 1-5 year mortality after a first apparent myocardial infarction in men

	**All men**			**Men 66-79 years**			**Men 80+ years**		
	**RR or HRR**	**95% Conf int**	**P value**	**RR or HRR**	**95% Conf int**	**P value**	**RR or HRR**	**95% Conf int**	**P value**
In Hospital Mortality									
N (Events)	7,929	(1059)		5,314	(527)		2,615	(532)	
Ed < HS	1.07	(0.87, 1.31)	0.544	1.06	(0.78, 1.43)	0.720	1.09	(0.82, 1.43)	0.558
Ed HS < BS	1.02	(0.83, 1.26)	0.819	1.04	(0.77, 1.40)	0.812	1.01	(0.76, 1.34)	0.955
Zip Inc q1	1.06	(0.84, 1.33)	0.636	1.08	(0.78, 1.49)	0.648	1.03	(0.74, 1.42)	0.875
Zip Inc q2	1.04	(0.85, 1.27)	0.707	1.03	(0.77, 1.39)	0.831	1.05	(0.80, 1.36)	0.739
Zip Inc q3	1.14	(0.95, 1.38)	0.165	1.19	(0.90, 1.57)	0.227	1.10	(0.86, 1.42)	0.442
Zip Inc q4	1.04	(0.87, 1.25)	0.659	0.92	(0.69, 1.21)	0.538	1.17	(0.92, 1.49)	0.198
Discharge - 365 day Mortality									
N (Events)	6,850	(1377)		4,776	(709)		2,074	(668)	
Ed < HS	1.00	(0.85, 1.17)	0.953	0.99	(0.78, 1.25)	0.918	1.00	(0.80, 1.25)	0.978
Ed HS < BS	0.97	(0.82, 1.14)	0.685	0.90	(0.71, 1.14)	0.374	1.04	(0.83, 1.31)	0.714
Zip Inc q1	*1.24*	*(1.04, 1.48)*	*0.016*	1.15	(0.88, 1.49)	0.303	*1.31*	*(1.03, 1.67)*	*0.026*
Zip Inc q2	1.07	(0.92, 1.26)	0.379	0.92	(0.72, 1.17)	0.486	*1.25*	*(1.02, 1.54)*	*0.031*
Zip Inc q3	1.03	(0.88, 1.21)	0.709	0.95	(0.75, 1.20)	0.663	1.13	(0.92, 1.39)	0.254
Zip Inc q4	1.15	(0.99, 1.33)	0.072	1.05	(0.84, 1.31)	0.669	*1.25*	*(1.03, 1.52)*	*0.027*
366 days - 5 year Mortality									
N (Events)	5,473	(1518)		4,067	(901)		1,406	(617)	
Ed < HS	1.21	(0.99, 1.48)	0.066	1.37	(1.06, 1.76)	0.017	0.99	(0.71, 1.40)	0.969
Ed HS < BS	*1.27*	*(1.04, 1.55)*	*0.021*	*1.32*	*(1.03, 1.69)*	*0.030*	1.16	(0.82, 1.64)	0.393
Zip Inc q1	*1.30*	*(1.07, 1.59)*	*0.009*	1.30	(1.00, 1.69)	0.052	1.33	(0.98, 1.81)	0.068
Zip Inc q2	1.03	(0.86, 1.24)	0.739	1.07	(0.85, 1.34)	0.576	0.99	(0.73, 1.33)	0.921
Zip Inc q3	0.95	(0.80, 1.13)	0.588	1.03	(0.83, 1.28)	0.785	0.86	(0.65, 1.14)	0.306
Zip Inc q4	1.13	(0.96, 1.33)	0.146	1.07	(0.87, 1.32)	0.516	1.25	(0.97, 1.61)	0.091

*Abbreviations: RR* Relative Risk (In-hospital and Discharge - 365 day mortality), *HRR* Hazard Rate Ratio (1-5 year mortality), *Ed* < HS Education less than a high school diploma, *Ed* HS < BS At least a high school diploma, but no college degree.

Adjusted for Age, race, hx of hypertension, hx of diabetes, hx of lipidemia, hx of CHD, hx of stroke, and coronary revascularization (PCI or CABG) during hospitalization, year of MI.

Education referent group is college degree or more. Zip code median income referent group is quintile 5 (highest median income).

Italicized associations, confidence intervals, and p-values denote those significant at alpha<0.05.

### Recurrence

Neither individual level education or zip code level median income were associated with recurrence in women (Table 4); however, education approached statistical significance with long-term recurrence in women 80+ years of age (HRR: < HS v College Degree + =1.52, P = .064). Contrary to expectations, zip code level median income in quintile 4 was associated with lower long-term recurrence (Q4 v Q5 = 0.65; P = 0.009), a potential false positive since all other associations were non-significant or above 1.0.

**Table 4:** Association of education, and zip code median income quintile with recurrent myocardial infarction or fatal CHD within 1 year or 1-5 years in women surviving 28 days after a first apparent myocardial infarction

	**All women**			**Women 66-79 years**			**Women 80+ years**		
	**HRR**	**95% Conf int**	**P value**	**HRR**	**95% Conf int**	**P value**	**HRR**	**95% Conf int**	**P value**
Recurrent MI/Fatal CHD (29 days-365 days)									
N (Events)	6,528	(889)		3,646	(375)		2,882	(514)	
Ed < HS	1.07	(0.77, 1.48)	0.698	1.40	(0.79, 2.48)	0.245	0.97	(0.65, 1.44)	0.869
Ed HS < BS	1.01	(0.73, 1.40)	0.957	1.44	(0.82, 2.55)	0.206	0.87	(0.58, 1.31)	0.504
Zip Inc q1	1.02	(0.77, 1.33)	0.914	0.89	(0.59, 1.32)	0.555	1.11	(0.77, 1.60)	0.580
Zip Inc q2	1.09	(0.86, 1.39)	0.465	0.91	(0.64, 1.29)	0.602	1.24	(0.90, 1.71)	0.198
Zip Inc q3	1.04	(0.83, 1.30)	0.740	0.87	(0.62, 1.20)	0.394	1.19	(0.88, 1.60)	0.263
Zip Inc q4	1.00	(0.80, 1.25)	0.983	0.81	(0.58, 1.14)	0.228	1.17	(0.87, 1.59)	0.296
Recurrent MI/Fatal CHD (366 days-5 years)									
N (Events)	4,681	(833)		2,837	(402)		1,844	(431)	
Ed < HS	1.36	(0.99, 1.88)	0.060	1.19	(0.76, 1.88)	0.445	1.52	(0.98, 2.37)	0.064
Ed HS < BS	1.17	(0.85, 1.62)	0.335	0.92	(0.58, 1.44)	0.700	1.46	(0.94, 2.27)	0.096
Zip Inc q1	0.80	(0.61, 1.05)	0.104	0.78	(0.52, 1.17)	0.226	0.82	(0.57, 1.19)	0.300
Zip Inc q2	0.97	(0.77, 1.22)	0.816	1.06	(0.77, 1.46)	0.717	0.89	(0.65, 1.23)	0.493
Zip Inc q3	0.88	(0.71, 1.09)	0.235	0.88	(0.64, 1.20)	0.421	0.89	(0.66, 1.20)	0.429
Zip Inc q4	*0.70*	*(0.56, 0.87)*	*0.002*	0.75	(0.54, 1.03)	0.072	*0.65*	*(0.47, 0.90)*	*0.009*

*Abbreviations: HRR* Hazard Rate Ratio, *Ed* < HS Education less than a high school diploma, *Ed* HS < BS At least a high school diploma, but no college degree.

Adjusted for Age, race, hx of hypertension, hx of diabetes, hx of lipidemia, hx of CHD, hx of stroke, and coronary revascularization (PCI or CABG) during hospitalization, year of MI.

Education referent group is college degree or more. Zip code median income referent group is quintile 5 (highest median income).

Italicized associations, confidence intervals, and p-values denote those significant at alpha<0.05.

As shown in Table 5, individual level education was associated with long term recurrence (1-5 years) in men 66-79 years of age (HRR: < HS v College Degree + =1.68, P = .004). No association was found for zip code level median income with short or long-term recurrence in men 66-79 years of age; however, in men 80+ years of age, the lowest quintile of zip code level median income was associated with increased short term recurrence (Q1 v Q5 = 1.72, P = 0.009).

**Table 5:** Association of education, and zip code median income quintile with recurrent myocardial infarction or fatal CHD within 1 year or 1-5 years in men surviving 28 days after a first apparent myocardial infarction

	**All men**			**Men 66-79 years**			**Men 80+ years**		
	**HRR**	**95% Conf int**	**Pvalue**	**HRR**	**95% Conf int**	**P value**	**HRR**	**95% Conf int**	**P value**
Recurrent MI/Fatal CHD (29 days-365 days)									
N (Events)	6,493	(832)		4,584	(484)		1,909	(348)	
Ed < HS	1.01	(0.79, 1.30)	0.908	1.12	(0.83, 1.52)	0.455	0.89	(0.59, 1.35)	0.581
Ed HS < BS	0.80	(0.62, 1.02)	0.076	0.74	(0.54, 1.00)	0.050	0.91	(0.59, 1.40)	0.681
Zip Inc q1	1.28	(0.99, 1.66)	0.065	1.03	(0.73, 1.46)	0.864	*1.72*	*(1.14, 2.57)*	*0.009*
Zip Inc q2	1.20	(0.96, 1.52)	0.113	1.13	(0.84, 1.52)	0.403	1.30	(0.90, 1.89)	0.162
Zip Inc q3	0.90	(0.71, 1.14)	0.393	0.82	(0.61, 1.11)	0.200	1.04	(0.71, 1.51)	0.857
Zip Inc q4	1.13	(0.91, 1.41)	0.257	0.98	(0.74, 1.29)	0.886	1.38	(0.98, 1.95)	0.064
Recurrent MI/Fatal CHD (366 days-5 years)									
N (Events)	4,778	(774)		3,602	(494)		1,176	(280)	
Ed < HS	*1.49*	*(1.11, 2.00)*	*0.008*	*1.68*	*(1.18, 2.41)*	*0.004*	1.18	(0.71, 1.96)	0.532
Ed HS < BS	*1.39*	*(1.04, 1.86)*	*0.027*	*1.46*	*(1.02, 2.08)*	*0.038*	1.32	(0.79, 2.21)	0.292
Zip Inc q1	1.24	(0.93, 1.65)	0.151	1.22	(0.84, 1.78)	0.288	1.26	(0.78, 2.03)	0.342
Zip Inc q2	1.13	(0.88, 1.45)	0.329	1.19	(0.88, 1.62)	0.262	1.04	(0.68, 1.60)	0.857
Zip Inc q3	1.00	(0.79, 1.27)	0.978	1.01	(0.75, 1.36)	0.956	1.01	(0.68, 1.51)	0.952
Zip Inc q4	1.09	(0.86, 1.37)	0.482	0.99	(0.74, 1.33)	0.942	1.31	(0.89, 1.92)	0.172

*Abbreviations: HRR* Hazard Rate Ratio, *Ed* < HS Education less than a high school diploma, *Ed* HS < BS At least a high school diploma, but no college degree.

Adjusted for Age, race, hx of hypertension, hx of diabetes, hx of lipidemia, hx of CHD, hx of stroke, and coronary revascularization (PCI or CABG) during hospitalization, year of first MI.

Education referent group is college degree or more.

Zip code median income referent group is quintile 5 (highest median income).

Italicized associations, confidence intervals, and p-values denote those significant at alpha<0.05.

## Discussion

In the linked NLMS-Medicare sample, lower individual level education was associated with increased mortality in women and men 1-5 years after a first apparent MI. Education less than a high school diploma was associated with a 61% increase in 1-5 year mortality in women 66-79 years of age and a 37% increase in 1-5 year mortality in men 66-79 years of age when compared to those with a college degree or more. Education below a high school diploma was also associated with a 68% increase in 1-5 year recurrence in men 66-79 years of age. While associations were largely limited to persons 66-79 years of age, there was some evidence for an association of education with long-term recurrence in older (80+ years) women. Median zip code level income was inconsistently associated with mortality and recurrence. Median zip code income associations with mortality and recurrence were observed only in older (80+ years) men. Positive associations were found with discharge-1 year mortality and 28 day-1 year recurrence. Approximately 25% of the sample resided in zip codes that were in the highest quintile of median income, while less than 14% resided in zip codes in the lowest quintile of median income substantiating, circumstantially, that surviving long enough to reach Medicare may be linked to area SES [5].

There is limited data concerning area or individual socioeconomic status with post MI outcomes in a Medicare population. In a large Medicare based study of over 130,000 MIs ascertained in 1994-1996, Rao et al. found that relative to those in the highest decile of zip code income, those in the lowest decile of income were at 25% greater risk of 30 day mortality and 14% greater risk of 1 year mortality [13]. These risks are comparable to those found in the present study in women for in-hospital mortality and in men for discharge to 1 year mortality. In a study of 10,557 Medicare beneficiaries newly diagnosed with at least one condition (stroke, MI, heart failure, hip fracture or cancer) in 1993, Wen and Christakis utilized Census data and a social survey of neighborhood residents to examine mortality associations in different sociodemographic groups. Neighborhood characteristics included zip code measures of affluence, poverty, and education. Measures of the social environment were derived from the Project on Human Development in Chicago Neighborhoods Community Survey, and poverty status was based on an indicator for Medicaid recipient. Wen and Christakis found that zip code SES and social environment were associated with mortality to the extent that a 1 standard deviation decrease in either index was roughly equivalent to an extra year of life, but primarily among the ‘non-poverty’ group [14].

Studies in the US with a broader age range have generally found a lack of association with individual level education or income. In a study of 2,142 acute MIs from the PREMIER registry, Bernheim et al found that after adjustment, neither household income nor education was associated with one year survival [19], and after a follow-up of 2 years, Alter et al found that among 3,407 patients hospitalized with acute MI in Ontario, Canada, the association of income with mortality was no longer significant after adjustment for conventional risk factors and preexisting cardiovascular events [20]. However, area SES has generally been positively associated with mortality in studies with a broader age range. Using multi-level proportional hazards models, Tonne et al found in the Worcester Massachusetts Heart Attack Study that among 3,423 acute MI cases, those in Census tracts with the highest income or education had significantly better survival [21], and among 705 acute MIs in Olmsted county, Minnesota, lower Census tract income was associated with mortality while individual level education was not [22]. Likewise, after an average follow-up of three years, Horne et al found that zip code level median income was associated with a combined endpoint of death/MI among 3,410 Salt Lake City patients with angiographically defined coronary artery disease [23].

The Medicare program has been shown to reduce differentials in the control of key cardiovascular risk factors such as blood pressure, glucose, and cholesterol [24], but despite these gains, there are potential mechanisms by which all persons may not benefit equally. Residing in more disadvantaged Census tracts has been associated with a longer pre-hospital delay [13,25], and lower likelihood of being treated at hospitals with catheterization facilities [13] potentially impacting directly the in-hospital prognosis. Survival at high volume hospitals has been shown to be better when compared to lower volume hospitals [26], but it is unclear if a correlation exists between use of high volume hospitals and area characteristics.

Individual level SES has previously been associated with cardiac rehabilitation in a Medicare population [27], and among survivors of MI, those with lower individual income or education were shown to be more likely to smoke, less likely to exercise, and more likely to consume alcohol [28] potentially impacting the longer term prognosis. Associations were more pronounced for income rather than education however, suggesting that income may detect stronger mortality gradients than education in an elderly population.

The NLMS-Medicare dataset offers the advantage of a large, nationally representative sample with self-reported measures of race and education. The relatively large size also permitted stratifying the sample by subgroups of interest, particularly sex, age and time since the initial MI. However, there are limitations that should be acknowledged. While the previous year’s medical history was used to define covariates, we were unable to account for some potential confounders such as hospital characteristics, smoking or medications. Interview dates for the ASEC survey could have been as many as 20 years prior to the index MI and given the varying lengths of time, we were limited to education as the sole indicator of individual SES. Individual SES is a multi-dimensional trait and characteristics such as income or social networks may be more reflective of socio-economic status in the elderly [29,30]. While geographic subdivisions such as Census tract or block define residential areas for the purpose of statistical reporting, zip codes are defined by the postal service as a means of efficiently delivering the mail and can thus change over time or be too heterogeneous to adequately reflect the neighborhood of an individual; however, in an incident MI case-control study in Washington state, the association of area characteristics such as median income or education with mortality were not found to be sensitive to the neighborhood definition used (1 km buffer, block group, census tract, zip code) [31].

## Conclusions

Area and individual socioeconomic characteristics acted independently on survival and recurrence post-MI among Medicare beneficiaries, and acted differentially with respect to time since the initial MI. With the exception of older men, area SES was largely not associated with survival or recurrence. Individual level education tended to impact longer term prognosis in both men and women. Future studies on differentials in survival and recurrence are needed to further our understanding of what barriers to immediate care may exist for persons residing in disadvantaged areas and on methods for improving access to longer term care.

## Competing interests

The authors have no actual or potential conflicts of interests.

## Authors’ contributions

SAC conceived the study, carried out the analysis and prepared the manuscript draft, NJJ collected the data and carried out the Medicare linkage and provided critical comments, JKH participated in analysis and provided critical comments on the manuscript, PDS participated in the design of the study and provided critical comments on the manuscript. All authors read and approved the final manuscript.

## Pre-publication history

The pre-publication history for this paper can be accessed here:

http://www.biomedcentral.com/1471-2458/14/705/prepub

## Supplementary Material

Additional file 1 SurvivalRecurrenceAfterMI_Medicare.pdf, supplementary tables with sensitivity results derived from re-weighting the Medicare sample to account for the probability of linkage to Medicare. Click here for file
