# Supplementary material for: Individual education, area income, and mortality and recurrence of myocardial infarction in a Medicare cohort: the National Longitudinal Mortality Study
Source: BMC Public Health. 2014 Jul 9;14:705. doi: 10.1186/1471-2458-14-705 (PMC4227052; doi:10.1186/1471-2458-14-705)
Supplement: Additional file 1 — SurvivalRecurrenceAfterMI_Medicare.pdf, supplementary tables with sensitivity results derived from re-weighting the Medicare sample to account for the probability of linkage to Medicare. [file 1471-2458-14-705-S1.pdf]

Supplement Table 1 – Re-weighted association of education, and zip code median income quintile with in-hospital, discharge to 1 year, and 1-5 year mortality after a first apparent myocardial infarction in women.

	All Women			Women 66-79 years			Women 80+ years		
	RR or	95% Conf Int	Pvalue	RR or	95% Conf Int	Pvalue	RR or	95% Conf Int	Pvalue
In Hospital Mortality									
N (Events)	8,043	(1153)		4,223	(454)		3,820	(699)	
Ed < HS	1.04	(0.80 , 1.34)	0.791	0.85	(0.55 , 1.32)	0.477	1.14	(0.83 , 1.58)	0.412
Ed HS < BS	1.06	(0.82 , 1.37)	0.644	0.77	(0.50 , 1.19)	0.241	1.28	(0.93 , 1.76)	0.125
Zip Inc q1	1.08	(0.88 , 1.33)	0.479	1.35	(0.97 , 1.88)	0.072	0.92	(0.69 , 1.21)	0.537
Zip Inc q2	1.00	(0.83 , 1.21)	1.000	1.15	(0.83 , 1.57)	0.399	0.93	(0.74 , 1.17)	0.540
Zip Inc q3	0.93	(0.77 , 1.13)	0.483	0.95	(0.67 , 1.34)	0.752	0.93	(0.74 , 1.17)	0.536
Zip Inc q4	0.89	(0.74 , 1.07)	0.212	0.82	(0.60 , 1.12)	0.217	0.95	(0.76 , 1.19)	0.664
Discharge - 365 day Mortality									
N (Events)	6,852	(1392)		3,760	(484)		3,092	(908)	
Ed < HS	1.06	(0.85 , 1.32)	0.616	1.26	(0.81 , 1.95)	0.307	1.01	(0.78 , 1.30)	0.936
Ed HS < BS	0.92	(0.73 , 1.15)	0.449	0.98	(0.63 , 1.52)	0.926	0.92	(0.71 , 1.19)	0.531
Zip Inc q1	1.04	(0.87 , 1.25)	0.662	0.89	(0.63 , 1.26)	0.515	1.10	(0.90 , 1.36)	0.350
Zip Inc q2	1.04	(0.89 , 1.22)	0.605	0.94	(0.69 , 1.27)	0.672	1.08	(0.90 , 1.30)	0.389
Zip Inc q3	1.02	(0.87 , 1.20)	0.765	0.97	(0.72 , 1.30)	0.829	1.04	(0.86 , 1.26)	0.672
Zip Inc q4	1.01	(0.86 , 1.18)	0.884	0.88	(0.64 , 1.20)	0.415	1.08	(0.90 , 1.29)	0.421
366 days - 5 year Mortality									
N (Events)	5,460	(729)		3,276	(900)		2,184	(1518)	
Ed < HS	1.26	(0.99 , 1.62)	0.064	**1.83**	**(1.17 , 2.86)**	**0.008**	0.99	(0.74 , 1.34)	0.971
Ed HS < BS	1.13	(0.89 , 1.45)	0.317	**1.56**	**(1.01 , 2.42)**	**0.046**	0.93	(0.68 , 1.26)	0.628
Zip Inc q1	0.94	(0.77 , 1.16)	0.561	1.05	(0.78 , 1.42)	0.762	0.88	(0.67 , 1.16)	0.351
Zip Inc q2	0.87	(0.72 , 1.05)	0.140	0.92	(0.69 , 1.22)	0.551	0.85	(0.67 , 1.09)	0.200
Zip Inc q3	0.86	(0.72 , 1.02)	0.086	0.99	(0.77 , 1.28)	0.959	**0.77**	**(0.61 , 0.98)**	**0.034**
Zip Inc q4	0.97	(0.81 , 1.15)	0.697	1.04	(0.80 , 1.36)	0.768	0.93	(0.73 , 1.18)	0.562

Abbreviations: RR = Relative Risk (In-hospital and Discharge - 365 day mortality); HRR = Hazard Rate Ratio (1-5 year

Adjusted for Age, race, hx of hypertension, hx of diabetes, hx of lipidemia, hx of CHD, hx of stroke, and coronary revascularization (PCI or CABG) during hospitalization, year of MI.

Education referent group is college degree or more.

Zip code median income referent group is quintile 5 (highest median income).

Supplement Table 2 – Re-weighted association of education, and zip code median income quintile with in-hospital, discharge to 1 year, and 1-5 year mortality after a first apparent myocardial infarction in men.

	All Men			Men 66-79 years			Men 80+ years		
	RR or HRR	95% Conf Int	Pvalue	RR or HRR	95% Conf Int	Pvalue	RR or HRR	95% Conf Int	Pvalue
In Hospital Mortality									
N (Events)	7,929	(1059)		5,314	(527)		2,615	(532)	
Ed < HS	1.04	(0.84, 1.29)	0.695	1.02	(0.74, 1.40)	0.916	1.07	(0.81, 1.42)	0.627
Ed HS < BS	1.03	(0.83, 1.27)	0.818	1.05	(0.76, 1.43)	0.778	0.99	(0.74, 1.32)	0.952
Zip Inc q1	1.05	(0.83, 1.34)	0.664	1.05	(0.74, 1.48)	0.796	1.06	(0.76, 1.47)	0.751
Zip Inc q2	1.03	(0.84, 1.26)	0.798	1.02	(0.75, 1.39)	0.883	1.04	(0.79, 1.37)	0.785
Zip Inc q3	1.19	(0.98, 1.44)	0.079	1.31	(0.99, 1.74)	0.061	1.09	(0.84, 1.41)	0.501
Zip Inc q4	1.05	(0.87, 1.27)	0.605	0.93	(0.69, 1.25)	0.616	1.19	(0.92, 1.52)	0.183
Discharge - 365 day Mortality									
N (Events)	6,850	(1377)		4,776	(709)		2,074	(668)	
Ed < HS	1.00	(0.84, 1.18)	0.959	0.99	(0.78, 1.27)	0.952	0.99	(0.79, 1.25)	0.958
Ed HS < BS	0.96	(0.81, 1.14)	0.612	0.92	(0.72, 1.17)	0.482	0.99	(0.79, 1.26)	0.962
Zip Inc q1	1.18	(0.99, 1.42)	0.068	1.05	(0.80, 1.38)	0.741	**1.32**	**(1.03, 1.69)**	**0.028**
Zip Inc q2	1.03	(0.88, 1.22)	0.694	0.89	(0.69, 1.15)	0.384	1.20	(0.97, 1.48)	0.098
Zip Inc q3	1.00	(0.85, 1.18)	0.998	0.91	(0.72, 1.16)	0.455	1.10	(0.89, 1.38)	0.375
Zip Inc q4	1.07	(0.92, 1.25)	0.394	0.98	(0.78, 1.23)	0.858	1.16	(0.94, 1.42)	0.170
366 days - 5 year Mortality									
N (Events)	5,473	(1518)		4,067	(901)		1,406	(617)	
Ed < HS	1.22	(0.98, 1.51)	0.071	**1.36**	**(1.04, 1.78)**	**0.023**	0.99	(0.69, 1.43)	0.966
Ed HS < BS	**1.27**	**(1.03, 1.57)**	**0.027**	**1.32**	**(1.02, 1.71)**	**0.035**	1.15	(0.79, 1.66)	0.471
Zip Inc q1	**1.27**	**(1.02, 1.57)**	**0.029**	1.29	(0.98, 1.71)	0.071	1.23	(0.89, 1.71)	0.205
Zip Inc q2	0.95	(0.78, 1.15)	0.571	0.98	(0.77, 1.25)	0.902	0.91	(0.66, 1.24)	0.542
Zip Inc q3	0.91	(0.76, 1.09)	0.309	0.98	(0.78, 1.23)	0.851	0.83	(0.62, 1.12)	0.221
Zip Inc q4	1.04	(0.88, 1.24)	0.622	1.03	(0.82, 1.28)	0.806	1.10	(0.84, 1.46)	0.487

Abbreviations: RR = Relative Risk (In-hospital and Discharge - 365 day mortality); HRR = Hazard Rate Ratio (1-5 year

mortality); Ed < HS = Education less than a high school diploma; Ed HS <BS = At least a high school diploma, but no college degree

Adjusted for Age, race, hx of hypertension, hx of diabetes, hx of lipidemia, hx of CHD, hx of stroke, and coronary revascularization (PCI or CABG) during hospitalization, year of MI.

Education referent group is college degree or more.

Zip code median income referent group is quintile 5 (highest median income).

Supplement Table 3 – Re-weighted association of education, and zip code median income quintile with recurrent myocardial infarction or fatal CHD within 1 year or 1-5 years in women surviving 28 days after a first apparent myocardial infarction.

	All Women			Women 66-79 years			Women 80+ years		
	HRR	95% Conf Int	Pvalue	HRR	95% Conf Int	Pvalue	HRR	95% Conf Int	Pvalue
Recurrent MI/Fatal CHD (29 days-365 days)									
N (Events)	6,528	(889)		3,646	(375)		2,882	(514)	
Ed < HS	1.02	(0.73 , 1.43)	0.919	1.38	(0.78 , 2.45)	0.273	0.91	(0.60 , 1.36)	0.634
Ed HS < BS	0.96	(0.68 , 1.34)	0.796	1.40	(0.79 , 2.48)	0.244	0.82	(0.54 , 1.25)	0.351
Zip Inc q1	0.98	(0.74 , 1.30)	0.906	0.87	(0.57 , 1.33)	0.518	1.06	(0.73 , 1.54)	0.774
Zip Inc q2	1.06	(0.83 , 1.36)	0.624	0.90	(0.62 , 1.30)	0.573	1.19	(0.86 , 1.65)	0.300
Zip Inc q3	1.10	(0.87 , 1.38)	0.418	0.89	(0.63 , 1.26)	0.519	1.28	(0.94 , 1.74)	0.121
Zip Inc q4	1.02	(0.81 , 1.30)	0.842	0.79	(0.55 , 1.14)	0.207	1.24	(0.91 , 1.70)	0.174
Recurrent MI/Fatal CHD (366 days-5 years)									
N (Events)	4,681	(833)		2,837	(402)		1,844	(431)	
Ed < HS	1.33	(0.94 , 1.87)	0.107	1.03	(0.64 , 1.65)	0.915	**1.71**	**(1.07 , 2.72)**	**0.024**
Ed HS < BS	1.12	(0.79 , 1.59)	0.531	0.76	(0.47 , 1.22)	0.253	**1.63**	**(1.02 , 2.63)**	**0.042**
Zip Inc q1	0.76	(0.57 , 1.01)	0.055	0.72	(0.47 , 1.09)	0.120	0.80	(0.54 , 1.17)	0.254
Zip Inc q2	0.91	(0.72 , 1.16)	0.458	1.04	(0.74 , 1.47)	0.806	0.79	(0.57 , 1.10)	0.170
Zip Inc q3	0.86	(0.68 , 1.08)	0.200	0.86	(0.62 , 1.20)	0.379	0.87	(0.63 , 1.20)	0.383
Zip Inc q4	**0.66**	**(0.52 , 0.84)**	**<.001**	0.73	(0.52 , 1.02)	0.063	**0.59**	**(0.41 , 0.85)**	**0.005**

Abbreviations: HRR = Hazard Rate Ratio; Ed < HS = Education less than a high school diploma; Ed HS <BS = At least a high school diploma, but no college degree

Adjusted for Age, race, hx of hypertension, hx of diabetes, hx of lipidemia, hx of CHD, hx of stroke, and coronary revascularization (PCI or CABG) during hospitalization, year of MI.

Education referent group is college degree or more.

Zip code median income referent group is quintile 5 (highest median income).

Supplement Table 4 – Re-weighted association of education, and zip code median income quintile with recurrent myocardial infarction or fatal CHD within 1 year or 1-5 years in men surviving 28 days after a first apparent myocardial infarction.

	All Men			Men 66-79 years			Men 80+ years		
	HRR	95% Conf Int	Pvalue	HRR	95% Conf Int	Pvalue	HRR	95% Conf Int	Pvalue
Recurrent MI/Fatal CHD (29 days-365 days)									
N (Events)	6,493	(832)		4,584	(484)		1,909	(348)	
Ed < HS	1.02	(0.79, 1.32)	0.865	1.13	(0.82, 1.55)	0.449	0.88	(0.58, 1.34)	0.553
Ed HS < BS	0.80	(0.61, 1.03)	0.087	0.74	(0.54, 1.03)	0.076	0.89	(0.58, 1.36)	0.584
Zip Inc q1	1.27	(0.96, 1.69)	0.098	0.94	(0.64, 1.37)	0.740	**2.00**	**(1.32, 3.03)**	**0.001**
Zip Inc q2	1.24	(0.96, 1.59)	0.095	1.18	(0.85, 1.64)	0.314	1.29	(0.88, 1.90)	0.184
Zip Inc q3	0.91	(0.71, 1.17)	0.456	0.80	(0.58, 1.10)	0.169	1.10	(0.75, 1.63)	0.626
Zip Inc q4	1.14	(0.91, 1.44)	0.255	0.99	(0.74, 1.34)	0.964	1.40	(0.98, 2.00)	0.064
Recurrent MI/Fatal CHD (366 days-5 years)									
N (Events)	4,778	(774)		3,602	(494)		1,176	(280)	
Ed < HS	**1.50**	**(1.10, 2.04)**	**0.011**	**1.64**	**(1.13, 2.38)**	**0.010**	1.24	(0.71, 2.14)	0.451
Ed HS < BS	1.35	(0.99, 1.83)	0.056	1.37	(0.94, 1.98)	0.100	1.35	(0.77, 2.35)	0.291
Zip Inc q1	1.28	(0.93, 1.75)	0.130	1.23	(0.83, 1.84)	0.304	1.38	(0.83, 2.30)	0.220
Zip Inc q2	1.12	(0.86, 1.45)	0.410	1.13	(0.82, 1.55)	0.460	1.11	(0.70, 1.75)	0.667
Zip Inc q3	0.99	(0.77, 1.27)	0.910	0.98	(0.72, 1.34)	0.894	1.00	(0.65, 1.53)	1.000
Zip Inc q4	1.07	(0.84, 1.36)	0.602	0.97	(0.71, 1.31)	0.827	1.30	(0.86, 1.96)	0.209

Abbreviations: HRR = Hazard Rate Ratio; Ed < HS = Education less than a high school diploma; Ed HS <BS = At least a high school diploma, but no college degree

Adjusted for Age, race, hx of hypertension, hx of diabetes, hx of lipidemia, hx of CHD, hx of stroke, and coronary revascularization (PCI or CABG) during hospitalization, year of first MI.

Education referent group is college degree or more.

Zip code median income referent group is quintile 5 (highest median income).
